# Bilateral hypoglossal nerve stimulation for obstructive sleep apnea: a nonrandomized clinical trial

**DOI:** 10.5664/jcsm.11822

**Published:** 2025-11-01

**Authors:** B. Tucker Woodson, David T. Kent, Colin Huntley, Melyssa K. Hancock, Douglas J. Van Daele, Maurits S. Boon, Tod C. Huntley, Sam Mickelson, M. Boyd Gillespie, Maria V. Suurna, Ashutosh Kacker, Asim Roy, Stuart MacKay, Kirk P. Withrow, Raj C. Dedhia, Phillip Huyett, Clemens Heiser, Sylvie di Nicola, Fatima Makori, Olivier M. Vanderveken, Tapan A. Padyha, Ulysses J. Magalang, Eugene Chio, Eric J. Kezirian, Richard Lewis

**Affiliations:** ^1^ Medical College Wisconsin, Milwaukee, Wisconsin; ^2^ Vanderbilt Univ. Medical Center, Nashville, Tennessee; ^3^ Thomas Jefferson University, Philadelphia, Pennsylvania; ^4^ ENT & Allergy Associates of Florida, Boca Raton, Florida; ^5^ University of Iowa, Iowa City, Iowa; ^6^ Center for ENT & Allergy, Carmel, Indiana; ^7^ Advanced ENT Associates, Atlanta, Georgia; ^8^ The University of Tennessee Health Science Center, Memphis, Tennessee; ^9^ University of Miami Medical Center, Miami, Florida; ^10^ Weill Cornell Medicine, New York, New York; ^11^ Sleep Medicine Institute, Nebraska Ohio Medical University, Dublin, Ohio; ^12^ Head & Neck Clinic, Illawarra ENT, Wollongong, New South Wales, Australia; ^13^ University of Alabama at Birmingham, Birmingham, Alabama; ^14^ University of Pennsylvania, Philadelphia, Pennsylvania; ^15^ Harvard University, Mass Eye & Ear, Boston, Massachusetts; ^16^ Technische Universitat Munchen, München, Germany; ^17^ Inferential Premium Biometry, Paris, France; ^18^ Faculty of Medicine, Translational Neurosciences, Antwerp University Hospital and University of Antwerp, Antwerp, Belgium; ^19^ University of South Florida, Tampa, Florida; ^20^ The Ohio State University Wexner Medical Center, Columbus, Ohio; ^21^ Ohio ENT and Allergy Physicians, Columbus, Ohio; ^22^ Department of Head and Neck Surgery, David Geffen School of Medicine at University of California, Los Angeles, Los Angeles, California; ^23^ Perth Head & Neck Surgery, Perth, Western Australia, Australia

**Keywords:** obstructive sleep apnea, hypoglossal nerve stimulation, sleep surgery

## Abstract

**Study Objectives:**

To evaluate the safety and efficacy of a novel bilateral hypoglossal nerve stimulation (HNS_BL_) device for the treatment of obstructive sleep apnea.

**Methods:**

Adult patients with moderate-to-severe obstructive sleep apnea who refused, failed, or did not tolerate positive airway pressure therapy underwent implantation and nightly use of HNS_BL_. The coprimary endpoints at 12 months were (1) a minimum of 50% reduction in the 4% apnea-hypopnea index (AHI) from baseline with a final AHI of less than 20 events/h, and (2) a minimum of 25% reduction in the 4% oxygen desaturation index. Objective secondary endpoints included changes in mean AHI, oxygen desaturation index, and sleep time with blood oxygen saturation less than 90%. Self-reported secondary endpoints included changes in Epworth Sleepiness Scale, the short Functional Outcomes of Sleep Questionnaire score, the Symptoms of Nocturnal Obstruction and Related Events score, and bedpartner assessment of snoring.

**Results:**

HNS_BL_ was implanted in 113 participants. Eleven serious adverse events occurred in 10 (8.7%) participants. The coprimary endpoints were completed by 89 (77.4%) participants. AHI and oxygen desaturation index responses were achieved in 63.5% (73/115, *P* = .002) and 71.3% (82/115, *P* < .001), respectively. Secondary endpoint analysis revealed significant changes in mean AHI (−18.3 ± 11.8 events/h, *P* < .001), oxygen desaturation index (−17.7 ± 14.6 events/h, *P* < .001), and sleep time with blood oxygen saturation less than 90% (6.9 ± 10.7%, *P* < .001). Significant changes were observed in all secondary endpoints (*P* < .001).

**Conclusions:**

This pivotal clinical trial of HNS_BL_ demonstrated an acceptable safety profile with clinically significant improvements in obstructive sleep apnea severity and quality-of-life metrics. HNS_BL_ is a promising new treatment option for select patients with obstructive sleep apnea.

**Clinical Trial Registration:** Registry: ClinicalTrials.gov; Name: Dual-sided Hypoglossal NeRvE StimulAtion for the TreatMent of Obstructive Sleep Apnea (DREAM); URL: https://clinicaltrials.gov/study/NCT03868618; Identifier: NCT03868618.

**Citation:**

Woodson BT, Kent DT, Huntley C, et al. Bilateral hypoglossal nerve stimulation for obstructive sleep apnea: a nonrandomized clinical trial. *J Clin Sleep Med*. 2025;21(11):1883–1891.

**BRIEF SUMMARY**

**Current Knowledge/Study Rationale:** Bilateral hypoglossal nerve stimulation is a novel approach to treat patients who have failed, refused, or not tolerated positive airway pressure or positional therapy. Therapy efficacy and safety were assessed over 12 months in 115 participants in a prospective, multicenter clinical trial.

**Study Impact:** The coprimary outcomes documented significant treatment response rates for decrease in the apnea-hypopnea index (63.5%; 73/115, *P* = .002) and the oxygen desaturation index (71.3%; 82/115, *P* < .001). Eleven serious adverse events occurred in 10 (8.7%) participants of which 3 (2.6%) were device related, 5 (4.3%) were procedure related, and 3 (2.6%) were unrelated to the device or the procedure.

## INTRODUCTION

Obstructive sleep apnea (OSA) is a common disease characterized by recurrent episodes of upper airway obstruction during sleep.^1^ Moderate-to-severe OSA affects nearly a half-billion people worldwide,^2^ and has been associated with an increased risk of cardiovascular mortality and decreased quality of life.^1^^,^^3^^–^^5^ Positive airway pressure (PAP) is well established as the first-line treatment modality for OSA, but many patients struggle to maintain adherence due to myriad side effects.^1^^,^^6^^,^^7^

Numerous alternative treatment options for OSA have been developed, including neuromodulation therapies. Hypoglossal nerve stimulation (HNS) has become an attractive anatomy-sparing treatment option because it is clinically effective while avoiding much of the surgical morbidity associated with traditional pharyngeal and craniofacial surgeries.^8^^–^^10^ It alleviates upper airway obstruction by protruding the tongue to increase pharyngeal cross-sectional area at multiple levels.^11^^–^^13^ A unilateral HNS device consisting of 3 implanted components (an implantable pulse generator with stimulation and respiratory sensing leads) was approved by the United States Food and Drug Administration in 2014. Nevertheless, postapproval studies have documented that it may lose efficacy in supine sleep when the pharynx is typically most collapsible.^14^^,^^15^ Implantation of the device’s respiratory sensor between the intercostal muscles additionally carries the risk of pneumothorax,^16^ and depletion of the non-rechargeable power source requires repeated surgeries for replacement.

An externally-powered, leadless, bilateral HNS (HNS_BL_) device (Genio; Nyxoah SA, Mont-Saint-Guibert, Belgium) was designed to address existing deficiencies in available neuromodulation therapy for OSA.^17^ The single-component implant is novel in that it stimulates both hypoglossal nerves without the use of an implanted battery or respiratory sensor (**Figure S1** in the supplemental material). This pivotal study was a multicenter, prospective, nonrandomized, single-arm trial designed to assess the safety and efficacy of HNS_BL_.

## METHODS

The Dual-sided hypoglossal neRvE stimulAtion for the treatMent of OSA study is a 5-year, international, prospective, single-arm pivotal trial of the HNS_BL_ device. There is no comparison cohort. The published trial protocol adheres to consolidated standards of reporting trials (CONSORT) guidelines and was approved by the institutional review board or medical ethics committee of each participating center.^18^ It was designed and funded by the sponsor. Primary and secondary outcomes were assessed at baseline and 12-months postoperatively in a single cohort of implanted participants. The writing committee (first 3 authors) had full access to all study data. The data were source verified by independent monitors and analyzed by an independent statistician.

### Participants

Adult patients with sleep apnea with failure, intolerance, or refusal of PAP therapy were recruited at academic and community sleep centers. Informed consent was obtained prior to study initiation. Key inclusion criteria consisted of age 22–75 (inclusive), body mass index of less than or equal to 32 kg/m^2^, a screening apnea-hypopnea index (AHI) between 15–65 events/h (inclusive) with combined central and mixed apnea indices of less than 25% of the total AHI as scored by a central core laboratory, at least 55 minutes of supine sleep, and a lack of complete concentric collapse on drug-induced sleep endoscopy as scored by a single, central reviewer who was blinded to the site investigator’s interpretation of the exam. A complete list of study inclusion and exclusion criteria have been previously published.^18^

### Intervention

The Genio system consists of an implantable stimulator powered by an external controller that is worn on the submentum during sleep using a disposable adhesive patch.^17^ The external controller contains a rechargeable battery and microprocessor that delivers participant-specific stimulation parameters to the passive implanted component using radiofrequency energy. The external controller was placed in the submental area nightly via a disposable adhesive patch. Participants with beards were not excluded but were counseled that facial hair needed to be trimmed to enable the patch to adhere to the skin. Stimulation parameters were adjusted at study visits by clinical staff, including titration type 1 polysomnograms (PSGs). The external controller hardware and software were both upgradeable, and while stimulation parameters remained fixed for most patients between study visits, some participants received an upgraded controller during the course of the study that enabled them to change the amplitude of HNS_BL_ at home within a provided range using an associated smartphone application. The upgrade also allowed for amplitude changes in smaller increments as compared to the original activation chip (1% as opposed to 5%) and provided 10 additional steps in amplitude below the original activation chip’s starting amplitude.

Participants who met PSG, demographic, and anthropometric criteria underwent HNS_BL_ implantation, as detailed in the published study protocol.^18^ Screening and baseline PSG variables of interest were averaged across the 2 preoperative PSGs due to the natural night-to-night variability of AHI.^19^

HNS_BL_ therapy was activated 2 months after implantation and adjusted during sponsor-attended PSGs and at subsequent clinic visits based on participant feedback.^18^ Twelve months following surgery, PSG was performed at fixed therapeutic settings to assess full-night efficacy. PSG studies were considered valid if the participant achieved at least 4 hours of sleep with at least 55 minutes in both supine and nonsupine sleeping positions. If participants did not meet these criteria, sleep testing was repeated. Hypopneas were defined using a 30% decrease in airflow in association with at least a 4% drop in oxygen saturation. The 4% oxygen desaturation index (ODI) was comprised of all events resulting in at least a 4% drop in oxygen saturation. The study protocol dictated that participants who had not met study responder criteria or who were not considered optimized by the site investigator after the 6-month PSG could undergo 8- and 10-month PSGs instead of a single 9-month PSG.^18^ Ad libitum unscheduled visits for diagnostic evaluation and HNS_BL_ adjustment were permitted at the discretion of the study investigator to mimic real-world therapy management patterns.^20^

### Study outcomes

The coprimary endpoints at 12 months were (1) a minimum of 50% reduction in the 4% AHI from baseline with a final AHI of less than 20 events/h (Sher criteria),^21^ and (2) a minimum of 25% reduction in the 4% ODI.

Objective secondary outcomes included 12-month changes in ODI, AHI, and sleep time with blood oxygen saturation less than 90%. Self-reported secondary outcome measures included validated sleep-related quality-of-life questionnaires including the short Functional Outcomes of Sleep Questionnaire (score range 5–20, higher scores indicate greater sleep-related quality of life), the Epworth Sleepiness Scale (score range 0–24, higher scores indicate greater daytime sleepiness), and the Symptoms of Nocturnal Obstruction and Related Events (score range 0–25, higher scores indicate worsening effects of sleep on quality of life). Bedpartner reports of snoring (when applicable) were reported by participants on a categorical scale (no snoring, soft snoring, loud snoring, very intense snoring, or bedpartner/participant leaves room).

Functional swallowing, speech, and pain assessments were completed throughout the course of the study (**Figure S4** in the supplemental material). Participants were also asked to complete a 5-question HNS_BL_ satisfaction and tolerance questionnaire, and to keep a daily electronic or paper self-reported diary noting nights per week and hours per night of HNS_BL_ device use. Additional outcome assessments included participant adherence with therapy and the effects of HNS_BL_ on sleep staging and AHI stratified by sleeping position.

### Safety

Safety outcomes were assessed by recording the incidence of any procedure- or device-related nonserious or serious adverse event (SAE) using the MedDRA coding database during the study for a period of 12 months following surgery. Adverse events were documented following International Organization for Standardization 14155:2020 guidelines and were coded as serious if they resulted in death, life-threatening illness or injury, permanent impairment, or if they required in-patient hospitalization or intervention to prevent life-threatening complications.^22^ Site investigators additionally retained independent discretion to code an adverse event as serious even if they did not meet the aforementioned criteria. Safety data through the 12-month visit was adjudicated by an independent clinical events committee and reviewed by a data safety monitoring board. Safety outcomes continue to be monitored for device-related SAEs or deficiencies that occur beyond the 12-month visit and will be reported in future long-term follow-up publications.

### Statistics

Sample size was computed with an 80% statistical power and a 1-sided 2.5% level of significance, using a 1-sample exact binomial test to reject the null hypotheses of percentages of AHI and ODI responders at 12 months lower than 50% in favor of responder rates of 65% and 64%, respectively. Based on these assumptions, the minimum number of required evaluable participants was 98. A rate of 15% nonevaluable participants was estimated a priori, resulting in a minimum enrollment sample of 115 participants.

Statistical analyses were performed using SAS v9.4 (SAS Institute, Cary, NC). Descriptive analyses were performed for all safety and effectiveness assessments. Two-sided, 95% confidence intervals (Clopper-Pearson) were provided where relevant (exact Clopper-Pearson method for percentages). Statistical tests were 1-sided.

The overall significance level for this study was 2.5% (1-sided). The study was considered successful if the null hypotheses were rejected for both coprimary effectiveness endpoints. The worst-case imputation method was used for coprimary endpoint missing data with missing values categorized as nonresponse The null hypotheses were rejected if the *P*-value of the 1-sided exact binomial test comparing the responder percentages to the prespecified performance goal of 50% was < .025.

Hypotheses for the secondary outcomes were tested using a hierarchical strategy. The average of baseline visit value and screening value was used to calculate baseline PSG variables of interest (AHI, ODI, and sleep time with blood oxygen saturation less than 90%). For all other effectiveness and safety parameters, baseline value was defined as the last valid value prior to the implant procedure. Secondary outcomes were summarized with descriptive statistics and presented with 2-sided 95% confidence intervals. They were tested using a 1-sided 1-sample paired *t* test. Missing data were censored from the analyses. Safety outcomes were reported using descriptive statistics.

## RESULTS

**Figure 1:**
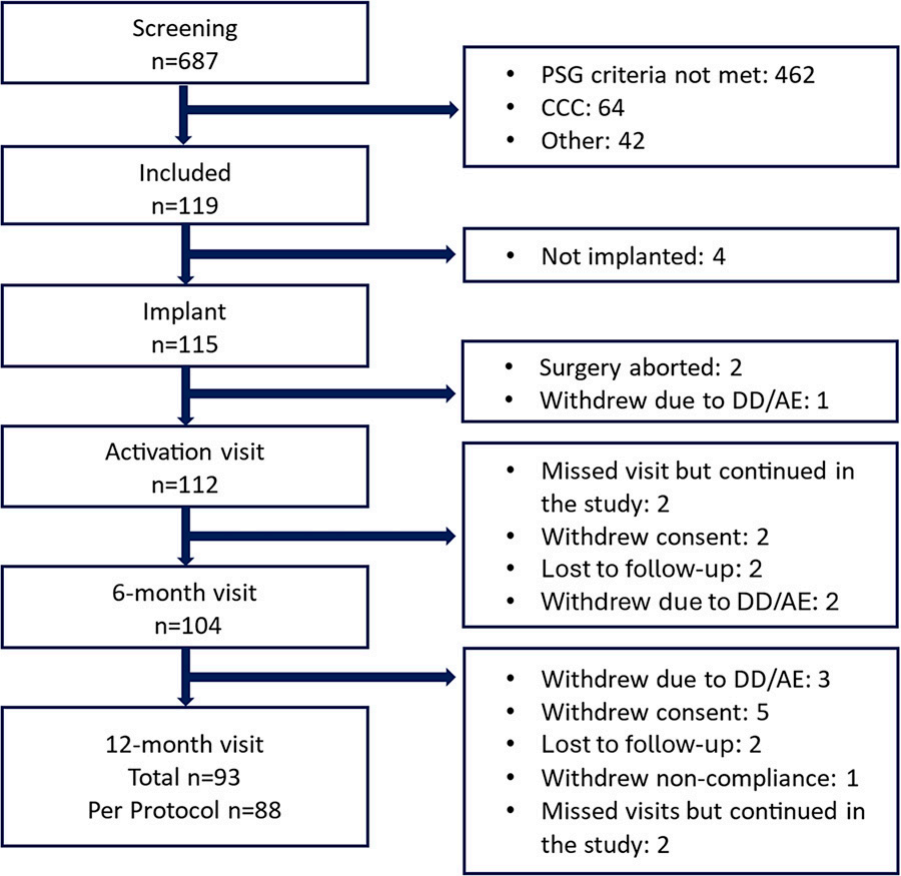
CONSORT diagram of study enrollment. Of 687 screened participants, 462 did not meet PSG inclusion criteria (see **Table S1** for details) or were excluded due to CCC of the palate during drug-induced sleep endoscopy. Six participants were withdrawn over the course of the study due to DDs or AEs that precluded further participation. Several other participants withdrew consent or were lost to follow-up, yielding 112 and 93 participants at the 6- and 12-month study visits, respectively, with 88 participants completing the study on a per-protocol basis. AE = adverse event, CCC = complete circumferential collapse, CONSORT = consolidated standards of reporting trials, DD = device deficiency, PSG = polysomnography.

A total of 687 participants signed informed consent and initiated screening procedures (**Figure 1**). Four hundred and sixty-two participants were excluded as they did not meet the PSG criteria (**Table S1** in the supplemental material), with most (n = 240) excluded for having only mild OSA (AHI < 15 events/h). Drug-induced sleep endoscopy excluded 64 participants with complete concentric collapse of the soft palate. Forty-two participants were excluded for being unwilling to comply with study requirements. Ultimately, 119 patients met inclusion criteria. The study design was capped at 115 participants eligible for implantation, leading to 4 implant-eligible participants being excluded after the 115th participant was implanted. The coprimary outcomes were assessed in 115 participants who underwent the implant procedure (**Table 1** and **Table S2** in the supplemental material). Most participants were middle aged, overweight, Caucasian males, some of whom had undergone prior surgery for sleep apnea, similar to other HNS study populations.^10^^,^^23^

**Table 1:** Baseline characteristics for study participants (n = 115) who underwent surgery for bilateral hypoglossal nerve stimulator implantation.

Demographic Characteristics	Mean ± SD (N = 115)	Median (Min; Max)
Age, year	56.8 ± 7.3	57 (36;71)
Male, sex	70.4% (81/115)	NA
Body mass index, kg/m^2^	28.5 ± 2.6	28.7 (21.7; 32.0)
Race: Caucasian	93.9% (108/115)	NA
**Polysomnographic Characteristics**	**Mean ± SD (n = 110)**	**Median (Min; Max)**
Apnea-hypopnea index, events/h	28.0 ± 11.5	25.6 (10.4; 59.1)
Apnea index, events/h	11.6 ± 9.5	9.7 (0; 48.4)
Hypopnea index, events/h	16.4 ± 9.0	15.4 (1.6; 50.6)
Oxygen desaturation index, events/h	27.0 ± 13.8	23.1 (9.8; 102.5)

Polysomnographic characteristics are presented for 110 implanted participants who underwent secondary outcome analyses. Max = maximum, Min = minimum, SD = standard deviation.

After the 6-month study visit, 56 (48.7%) participants were deemed not yet optimized with treatment and went on to have 8- and 10-month PSGs. Over the course of the study, 70 participants underwent at least 1 unscheduled study visit (**Table S3** in the supplemental material).

Twelve-month secondary outcomes were assessed in 110 participants, 89 of whom had data available for assessment of secondary PSG outcomes. The 12-month PSG was completed by 88/115 (76.5%) participants on a per-protocol basis (see “Participant attrition” below and **Table S4** in the supplemental material). Participant numbers for other self-reported secondary outcome assessments varied slightly based on whether they completed the requisite questionnaire at the associated clinical visit.

### Study outcomes

#### Primary outcomes

**Figure 2:**
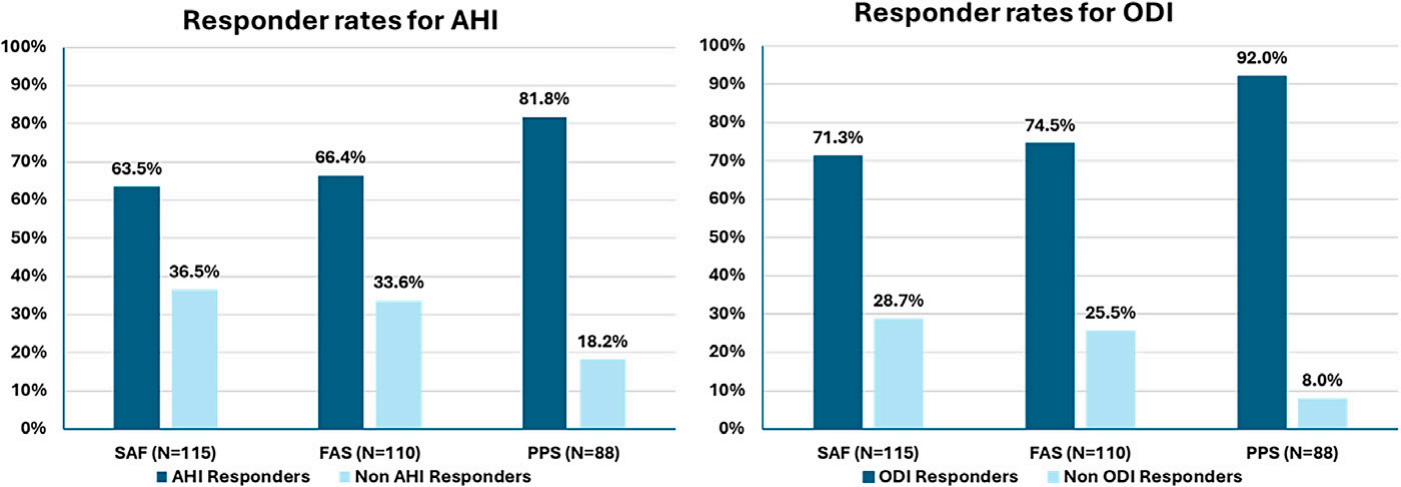
A sensitivity analysis of the coprimary endpoints at 12 months for AHI and ODI responders in the study. AHI responders achieved a minimum of 50% reduction in the 4% AHI from baseline with a final AHI of less than 20 events/h (Sher criteria),^21^ and ODI responders achieved a minimum of 25% reduction in the 4% ODI. All 115 participants who underwent surgery for bilateral hypoglossal nerve stimulator implantation underwent coprimary endpoint and SAF. A FAS was completed in 110 participants with available postoperative data. The study was completed per-protocol (PPS) by 88 participants. AHI = apnea-hypopnea index, FAS = full analysis of additional secondary outcomes, ODI = oxygen desaturation index, PPS = per-protocol set, SAF = safety outcome analysis.

The AHI and ODI coprimary outcome responder rates across all participants were 63.5% (73/115, *P* = .002) and 71.3% (82/115, *P* < .001), respectively using the worst-case imputation method with missing data considered a nonresponse. Sensitivity analyses of secondary outcomes were performed for the 115 participants (**Figure 2**).

#### Secondary outcomes

Twelve-month objective secondary outcomes were assessed in the 89 participants who completed the 12-month PSG. Clinically significant changes were observed in the AHI (−18.3 ± 11.8 events/h, *P* < .001), ODI (−17.7 ± 14.6 events/h, *P* < .001), and sleep time with blood oxygen saturation less than 90% (−6.9 ± 10.7% *P* < .001). All other secondary metrics also demonstrated clinically significant improvement (*P* < .001). Functional Outcomes of Sleep Questionnaire increased from 16.0 ± 2.3 to 18.2 ± 1.9. Epworth Sleepiness Scale decreased from 9.7 ± 5.6 to 6.2 ± 4.1, and Symptoms of Nocturnal Obstruction and Related Events decreased from 1.6 ± 0.9 to 0.6 ± 0.6 (**Figure S2**, **Figure S3**, and **Figure S4** in the supplemental material).

Snoring improved, with those reporting bedpartner leaving the room, very loud and loud snoring reduced from 83.5% at baseline to 30.4% at 12 months. At 12 months, participants’ satisfaction scores were reported as extremely satisfied (58.0%), somewhat satisfied (31.8%), somewhat dissatisfied (9.1%), and extremely dissatisfied (1.1%).

No changes in total sleep time or sleep stage distribution were observed in the exploratory analyses (*P* > .05). Total supine sleep time approached 2 hours but was reduced compared to baseline (*P* < .001; **Table 2**). Rapid eye movement (27.6 ± 24.2 to 8.3 ± 12.2 events/h) and supine AHI (48.9 ± 19.6 to 22.7 ± 19.9 events/h) were reduced at 12 months (*P* < .001; see **Figure S5** in the supplemental material). Mean per-participant percent reduction of total AHI was −66.1 ± 28.7% (median: 70.8%, *P* < .001), and mean per-participant percent reduction of supine AHI was −55.1 ± 36.5% (median: 66.6%, *P* < .001; see **Figure S6** and **Figure S7** in the supplemental material). In the per protocol group, AHI and ODI responder rates were 81.8% (72/88, *P* = .01) and 92.0% (81/88, *P* < .001), respectively.

**Table 2:** Secondary and additional endpoint outcomes.

Secondary Endpoints	Baseline Mean ± SD/Median	12 Month Mean ± SD/Median	Mean Change Mean ± SD	95% CI for Mean Change	*P*-Value
AHI, events/h	27.8 ± 11.5/24.3 (n = 89)	9.5 ± 9.4/6.8 (n = 89)	−18.3 ± 11.8	−20.8; −15.8	< .001
ODI, events/h	26.9 ± 14.4/22.8 (n = 89)	9.2 ± 9.6/5.9 (n = 89)	−17.7 ± 14.6	−20.8; −14.6	< .001
T90, minutes	11.9 ± 12.3/8.2 (n = 89)	5.0 ± 8.4/2.6 (n = 89)	−6.9 ± 10.7	−9.1; −4.7	< .001
Epworth Sleepiness Scale score	9.7 ± 5.6/10.0 (n = 87)	6.2 ± 4.1/5.0 (n = 87)	−3.5 ± 4.3	−4.4; −2.6	< .001
FOSQ-10 score	16.0 ± 2.9/16.4 (n = 88)	18.2 ± 1.9/19.0 (n = 88)	2.2 ± 2.8	1.6; 2.8	< .001
SNORE-25 score	1.6 ± 0.9/1.5 (n = 89)	0.6 ± 0.6/0.4 (n = 89)	−1.0 ± 0.8	−1.2; −0.9	< .001
**Additional Endpoints**	**Baseline Mean ± SD/Median**	**12 Month Mean ± SD/Median**	**Mean Change Mean ± SD**	**95% CI for Mean Change**	***P*-Value**
Supine sleep time, minutes	160.7 ± 56.3/148.9 (n = 89)	114.0 ± 55.1/98.5 (n = 89)	−46.6 ± 58.9	−59.1; −34.2	< .001
Nonsupine sleep time, minutes	202.4 ± 61.5/204.5 (n = 89)	248.7 ± 69.0/253.4 (n = 89)	46.4 ± 79.2	29.7; 63.0	< .001
REM AHI, events/h	23.3 ± 17.3/18.8 (n = 89)	8.9 ± 11.5/4.1 (n = 89)	−14.4 ± 19.2	−18.5; −10.3	< .001
NREM AHI, events/h	28.5 ± 13.2/25.3 (n = 89)	9.8 ± 10.4/6.2 (n = 89)	−18.8 ± 12.7	−21.4; −16.1	< .001
Supine AHI, events/h	49.4 ± 20.7/49.7 (n = 89)	22.7 ± 19.9/17.1 (n = 89)	−26.7 ± 20.6	−31.0; −22.4	< .001
Nonsupine AHI, events/h	11.8 ± 11.4/7.8 (n = 89)	5.2 ± 8.2/2.6 (n = 89)	−6.6 ± 11.5	−9.0; −4.2	< .001
Apnea index, events/h	11.6 ± 9.6/9.2 (n = 89)	3.9 ± 6.4/1.3 (n = 89)	−7.7 ± 10.2	−9.8; −5.6	< .001
Hypopnea index, events/h	16.3 ± 9.3/14.8 (n = 89)	5.7 ± 6.0/7.0 (n = 89)	−10.6 ± 8.7	−12.4; −8.8	< .001

AHI = apnea-hypopnea index, CI = confidence interval, FOSQ-10 = Functional Outcomes of Sleep Questionnaire, NREM = non-rapid eye movement, ODI = oxygen desaturation index, REM = rapid eye movement, SD = standard deviation, SNORE-25 = Symptoms of Nocturnal Obstruction and Related Events, T90 = percent of sleep time with blood oxygen saturation less than 90%.

### Safety outcomes

Ten (8.7%) participants experienced 11 SAEs of which 8 were device- and/or procedure-related (see **Table 3** and **Table S5** and **Table S6** in the supplemental material).

**Table 3:** Serious adverse events that occurred during the study, including bilateral hypoglossal nerve stimulation device- or implant procedure-related events.

Serious Adverse Events	m (n, %)
Unrelated to device and unrelated to implant procedure	3 (2, 1.7%)
Device-related and/or implant procedure-related	8 (8, 7.0%)
Device-related	3 (3, 2.6%)
Implant procedure-related	5 (5, 4.3%)

The same participant could have more than 1 event. m = number of events; n = number of participants with at least 1 event.

Five (4.3%) participants experienced procedure-related SAEs. One experienced epistaxis after nasal intubation, 1 experienced left bundle branch block postoperatively, and 1 had a hematoma of the implant site that required surgical drainage. Two (1.7%) participants experienced temporary hypoglossal nerve paralysis postoperatively (reported as dysphagia), even though no concerns for intraoperative injury were reported by the respective study site surgeons.

Three (2.6%) participants experienced serious adverse device events resulting in surgical management during the study. Of these, 2 participants with successful intraoperative testing experienced complete failure of hypoglossal nerve activation at the activation visit due to presumptive postoperative migration of the devices. Both underwent revision procedures, but scar tissue prevented successful repositioning and the implants were subsequently removed. Although neither of these migration events met International Organization for Standardization definitions for an SAE, the respective study site investigators independently elected to code them as such. The third participant experienced device extrusion into the floor of the mouth after an invasive dental procedure resulting in device explantation without further complications.

Seven (6.1%) other participants experienced non-SAEs involving diminished or complete loss of stimulation effect within 12 months of implantation. Of these, 2 (1.7%) were found to have malfunctioning devices which were electively replaced. Two (1.7%) other participants elected for device explantation after documented migration of the device. The cause for loss of stimulation could not be determined in the remaining 3 (2.6%), 1 of whom elected for removal of the device before the 12-month PSG after it could not be successfully replaced.

Ultimately, device- or procedure-related adverse events were experienced by 62.6% of participants with 59/210 (28.1%) nonserious events scored as moderate to severe in intensity. The majority (93.4%) of nonserious device- and procedure-related adverse events were self-limited and resolved within the first month of postoperative recovery (**Table S7** in the supplemental material).

### Device usage

Nightly HNS_BL_ usage was greater than 4 hours in more than 70% of nights in 84.3% (59/70) for participants completing diary entries in the 3 months preceding the 12-month visit. The device was used over 70% of the nights by 85.9% (61/71) of the participants.

### Participant attrition

As noted above, 88/115 (76.5%) of the participants undergoing implantation completed the 12-month PSG per protocol. While 1 of the remaining participants was withdrawn from the study by the site investigator for failure to maintain adherence with nightly therapy use, many of the remaining 26/115 (22.6%) participants missed or did not complete the 12-month PSG per protocol for other quantifiable reasons (see **Table S4** for per-participant details):

1. Surgery: Implantation was unsuccessful in 2 (1.7%) participants. One participant had a prominent ranine vein overlying one of the hypoglossal nerves and the study site surgeon was not comfortable ligating it to implant the HNS_BL_ device. In the second patient, tongue retrusion was observed intraoperatively with HNS_BL_ despite attempts to reposition the implant, ultimately necessitating device removal per the study protocol.
2. Regulatory adherence: At the request of regulatory monitors, 3 (2.6%) participants were voluntarily excluded by the sponsor from secondary analyses because clinic visits were shifted to a non-approved alternative site during the COVID-19 pandemic.
3. Adverse events or device deficiencies: 7 (6.1%) participants experienced adverse events or device deficiencies that prevented completion of the 12-month PSG per protocol (see “Safety outcomes,” above).
4. Protocol deviations: 1 (0.9%) participant did not sleep supine during the 12-month PSG for the required minimum duration of 55 minutes to meet protocol requirements. Three (2.6%) participants missed completing the 12-month PSG within the 1-month, per-protocol window for various personal reasons that interfered with scheduling.
5. Study exit: 4 (3.5%) participants were lost to follow-up. Six (5.2%) participants withdrew consent for reasons that were not disclosed by the participants. Withdrawal of consent prevented further data collection by study staff to identify reasons for withdrawal.

No statistically significant differences were noted in demographic, anthropometric, or PSG variables between those who completed the 12-month PSG and those who did not (*P* > .05; **Table S8** in the supplemental material).

## DISCUSSION

This pivotal clinical trial of HNS_BL_ demonstrated substantial reductions in OSA severity and symptoms in a population of continuous PAP-intolerant patients. The study reached both primary endpoints for therapy efficacy during the final assessment PSG at 12 months despite a protocol that mandated at least 1 hour of supine sleep, where OSA is typically most severe.^15^^,^^24^ Secondary endpoints and additional analyses yielded clinically meaningful improvements in quality-of-life and daytime sleepiness indices. Participants who completed the protocol demonstrated high treatment adherence rates at the 12-month visit and 89.8% indicated satisfaction with therapy. Taken together, the results document that battery-free, externally powered HNS_BL_ is a promising treatment option for well selected patients with moderate-to-severe OSA.

Existing evidence for HNS primarily originates from studies of unilateral treatment. Animal and human physiology studies previously demonstrated that effective unilateral therapy dilated the pharynx at multiple levels, including the velopharynx.^11^ Enrollment criteria for the previously approved unilateral device’s pivotal trial were similar to this one, but this trial additionally mandated at least an hour of supine (and nonsupine sleep during each PSG. A recent study of a United States Food and Drug Administration-approved unilateral device suggested that therapy efficacy diminishes during supine sleep when the airway is most collapsible.^14^^,^^15^ Nevertheless, effectiveness analyses of this HNS_BL_ device documented clinically significant reductions in supine AHI. Further clinical research will be required to ultimately validate this finding.

There are several functional differences between this HNS_BL_ device and currently available neuromodulation therapy for OSA. First, this is the only device designed to stimulate both hypoglossal nerves, protruding the entire oral tongue. A prior study of unilateral HNS therapy documented cross-innervation of the hypoglossal nerve in a minority of patients leading to activation of both genioglossus muscles.^25^ Those with bilateral activation experienced greater increases in retrolingual and retropalatal space than those with unilateral activation, which may explain the significant effect of HNS_BL_ observed during supine sleep in this study. Second, the battery-free HNS_BL_ device is powered by an external controller, obviating the need for repeat surgeries to replace depleted power sources. Third, this device uses an open-loop stimulation duty cycle strategy, eliminating the need for respiratory sensing lead hardware and reducing the complexity of implanted components.^16^ More extensive clinical data are ultimately required to ascertain whether there are meaningful clinical differences between HNS devices regarding safety, efficacy, and stimulation timing strategies.

This study was opened to enrollment just prior to the COVID-19 pandemic, where worldwide quarantine requirements and economic disruptions created significant challenges for health care systems.^26^^,^^27^ As a consequence, some of the participating surgeons were limited to virtual training and proctoring. One participant was not implanted due to a ranine vein overlying the hypoglossal nerve that the study site surgeon was not experienced with ligating, although this is commonly done for other HNS devices.^28^ Another participant was not implanted as tongue retraction was observed during intraoperative device testing, despite attempts at repositioning the implant. The study site surgeon did not report any unique variation in hypoglossal nerve anatomy intraoperatively and felt that he was still optimizing his HNS_BL_ implantation technique at the time of this case. He did not encounter this issue in further HNS_BL_ implants with grossly similar anatomy. Despite these issues, the device was successfully implanted in 113/115 (98.3%) of participants.

Several participants also required HNS_BL_ revision, replacement, or explantation for postoperative device migration or hardware failure, potentially related to technical errors or device damage during the implantation procedure, and 62.6% of participants experienced at least 1 adverse event. Most adverse events were self-limited and resolved within the first month of postoperative recovery, and the overall rate was comparable to the rate of procedure-related (57%) and device-related (67%) adverse events observed in a pivotal study of unilateral HNS. As with unilateral HNS, it is anticipated that there will be similar opportunities to refine aspects of the implantation procedure to reduce the incidence of postoperative adverse events as surgeons gain further experience with the device in clinical practice.^28^^–^^32^

Ultimately, 88/115 (76.5%) of participants undergoing HNS_BL_ surgical implantation completed the 12-month PSG on a per-protocol basis with a Sher responder rate of 72/88 (81.8%), which is comparable to or greater than prior publications assessing unilateral HNS during efficacy PSG evaluation.^10^^,^^23^^,^^33^ A more conservative analysis incorporating all participants excluded from the per-protocol dataset yielded a Sher responder rate of 73/115 (63.5%). Similarity to responder rates in other unilateral HNS trials cannot be statistically rejected (*P* ≥ .70),^10^ despite this trial requiring at least an hour of supine sleep during all PSGs. Further studies of HNS_BL_ in standard clinical practice are required to definitively assess real-world efficacy in comparison to unilateral HNS.

There are several limitations to the design and outcomes of this study. First, this clinical trial was designed to evaluate HNS_BL_ in a select group of continuous PAP-intolerant patients with moderate-to-severe OSA. The single-arm, open-label design did not directly compare HNS_BL_ to continuous PAP or other available therapies. Care should be taken against generalizing these results to patients with elevated AHI or body mass index, and further investigation is required to evaluate HNS_BL_ efficacy in patients with complete circumferential palatal collapse. Second, study participants were largely comprised of overweight, white male older adults, as is seen extensively throughout other sleep surgery cohorts.^34^ Further work is necessary to assess the generalizability of these results to females and individuals of other races, although available data suggest that they tend to respond comparably when treated with unilateral HNS therapy.^31^^,^^35^^,^^36^ Third, 27 participants (23.5%) did not complete the trial per protocol for a variety of reasons, and it is unknown whether a lower degree of attrition would have materially altered the observed per-protocol efficacy. The COVID-19 pandemic created significant logistical challenges for clinical research enterprises.^27^ Despite the attrition observed during this study, a supplemental analysis did not demonstrate any significant differences in demographic, anthropometric, or PSG characteristics between those who did and did not complete the study (**Table S8**), suggesting that attrition likely did not introduce systematic biases into the observed outcomes. Last, therapy adherence was self-reportedly assessed by participant self-reporting and may not accurately assess their true therapy utilization rates. While 61/70 (84.3%) of participants completing diary entries reported frequent use of HNS_BL_ in the 3 months preceding the 12-month PSG, it is unknown what adherence rates were in the remaining 19 participants who completed the PSG. As with any device-based therapy, failure to adequately use HNS_BL_ will reduce the overall effectiveness of therapy for patients. The current version of the HNS_BL_ software is capable of recording usage data, but this study protocol did not collect these data for analysis. Future assessments of HNS_BL_ will benefit from contrasting therapy adherence against PSG metrics of efficacy to best understand overall disease burden reduction.

This pivotal clinical trial investigated HNS_BL_ efficacy and safety in a select group of patients with moderate-to-severe OSA who were unaccepting of or unable to benefit from PAP therapy. The surgical procedure and device exhibited acceptable safety and side effects profiles, and the cohort experienced clinically significant improvements in objective measures of OSA severity and self-reported quality-of-life metrics.

## DISCLOSURE STATEMENT

The authors confirm that the manuscript has been seen and approved by all named authors. The study was conducted at the above-listed institutions. The study is sponsored by Nyxoah. The funder designed and conducted the study; collection of the data was also done by the funder. Management and analysis of the data was done by independent statisticians and verified by the publication committee, along with data interpretation. Preparation, review, or approval of the manuscript and the decision to submit the manuscript for publication was also performed by the publication committee. Dr. Woodson is a consultant for Nyxoah, Medtronic, Linguaflex, and Cryosa, is on the advisory board of Cryosa, received research funding from Nyxoah, Linguaflex, and Medtronic during the course of the study, and is coordinating investigator for DREAM. Dr. Kent is a consultant for Restera; is on the advisory board of Nyxoah; received research funding from Inspire Medical Systems, Nyxoah, Restera; inventor on US and international patents and applications owned by Vanderbilt University and licensed to Nyxoah SA; received research funding from National Heart, Lung, and Blood Institute (NHLBI) (R01HL161635). Dr. C. Huntley is a consultant for Inspire Medical Systems, Nyxoah, Avivomed; received research funding from Inspire Medical Systems, Nyxoah. Dr. Hancock is a consultant for Nyxoah; received research funding from Nyxoah. Dr. Van Daele received research funding from Inspire Medical Systems, Nyxoah, Checkpoint. Dr. Boon is an employee of Nyxoah. Dr. Mickelson is on the advisor board of Zelegent Inc.; received research funding from Nyxoah, Livanova. Dr. Gillespie is a consultant for HuMannity; is on the advisor board of CryOSA; received research funding from Inspire Medical Systems, Nyxoah. Dr. Suurna is a consultant for Nyxoah, Inspire Medical Systems; received research funding from Nyxoah, CryOSA; received honoraria from Nyxoah, Inspire Medical Systems. Dr. Kacker is a consultant for Sanofi, Genzyme; received research funding from Inspire Medical Systems, Nyxoah, Livanova. Dr. Roy received research funding from Nyxoah, Inspire, Takeda, Alkermes, Noctrix, Jazz, Harmony, Apnimed, Avadel, Signifier, Fisher Paykel, Amenity, Zoll/Respicardia; received honoraria from Jazz, Inspire, Harmony, Avadel; received speakership fees from Jazz, Inspire, Harmony, Avadel, Axsome. Dr. Mackay received research funding from National Health and Medical Research Council of Australia, Flinders University, and The Repat Foundation 2013 Prabha Seshadri Research Grant; non-financial support from Nyxoah; received grants from Garnett-Passe Rodney Williams Foundation, Illawarra Health and Medical Research Institute. Dr. Withrow is a consultant for Avivomed. Dr. Dedhia is on the advisory board of Lunair; received research funding from Inspire Medical Systems, Nyxoah, CryOSA. Dr. Huyett is a consultant for Inspire Medical Systems (educational grant); received research funding from Inspire Medical Systems, Nyxoah. Prof. Heiser is a consultant for Nyxoah; received research funding from Inspire Medical Systems, Nyxoah, Löwenstein Medizintechnik through Institution; received travel assistance from XM Consult, Inspire Medical Systems and Nyxoah; He is also the founder of Institute for Sleep Medicine. Prof. Vanderveken is a consultant for Inspire Medical Systems; received research funding from ProSomnus, Nyxoah, Inspire Medical Systems, Somnomed; receives a Senior Clinical Fellowship at FWO Research Foundation Flanders, Belgium. Dr. Padhya received research funding from Nyxoah. Dr. Magalang received research funding from NHLBI (R01HL175579); NHLBI (1 P01 HL160471); National Cancer Institute (R21CA276027); Department of Defense-Peer Reviewed Medical Research Program (PR212399). Dr. Chio was a consultant for Inspire Medical Systems and is a consultant for Nyxoah. Dr. Kezirian is a consultant for Nyxoah, huMannity Medtec, Berendo Scientific, Split Rock Scientific, is on the advisory board of Cryosa, and has received research funding from Inspire Medical Systems. Drs. Lewis, T. Huntley, Withrow, Ms. di Nicola and Ms. Makori declare no conflicts of interest.

## OPEN ACCESS

Copyright 2025 The Authors. This is an open access article, distributed under the Creative Commons Attribution 4.0 International License. Sharing and adaptation are permitted provided attribution to its original publication in the Journal of Clinical Sleep Medicine is made in accordance with the license.

## Supplemental Materials

10.5664/jcsm.11822 Supplemental Materials

## ABBREVIATIONS

AHI: apnea-hypopnea index
HNS: hypoglossal nerve stimulation
HNS_BL_: bilateral hypoglossal nerve stimulation
ODI: oxygen desaturation index
OSA: obstructive sleep apnea
PAP: positive airway pressure
PSG: polysomnogram
SAE: serious adverse event
