# Supplementary material for: Bilateral hypoglossal nerve stimulation for obstructive sleep apnea: a nonrandomized clinical trial
Source: J Clin Sleep Med. 2025 Nov 1;21(11):1883–91. doi: 10.5664/jcsm.11822 (PMC12582211; doi:10.5664/jcsm.11822)
Supplement: Supplementary file 1 [file jcsm.11822.sm001.pdf]

Criteria	n
AHI < 15 events/h	240
AHI > 65 events/h	4
Non-supine AHI > 10 events/h	153
Mixed + Central AHI > 25% total AHI	51
Insufficient sleep time (< 4 hours)	14
**TOTAL**	462

Table S1. Reasons for participant exclusion from the study based on polysomnography (PSG) exclusion criteria. AHI = apnea-hypopnea index. N = number of participants.

Demographic / Participant Characteristics	Mean ± SD (N = 115)	Median (Min; Max)
Age, year	56.8 ± 7.3	57 (36;71)
Male, gender	70.4% (81/115)	NA
Body Mass Index, kg/m^2	28.5 ± 2.7	28.7 (21.7; 32.0)
BP Systolic, mmHg	132.6 ± 16.5	SBP 131 (92; 188)
BP Diastolic, mmHg	79.9 ± 10.0	DBP 80 (53; 105)
Race: Caucasian	93.9% (108/115)	NA
**Previous Surgeries**	**n**	
Uvulopalatopharyngoplasty	17	
Hyoid Advancement	2	
**Medical History**	**n (%)**	
Hypertension	47 (40.9%)	
Gastroesophageal reflux disease (GERD)	34 (29.6%)	
Depression	28 (24.3%)	
Hypercholesterolemia	25 (21.7%)	
Hyperlipidemia	21 (18.3%)	
Anxiety	21 (18.3%)	
**OSA Characteristics**	**Mean ± SD (N = 110)**	**Median (Min; Max)**
Apnea-hypopnea index, events/h	28.0 ± 11.5	25.6 (10.4; 59.1)
Oxygen desaturation index, events/h	27.0 ± 13.8	23.1 (9.8; 102.5)

Table S2. Baseline characteristics for study participants (n=115) who underwent surgery for bilateral hypoglossal nerve stimulator implantation. Polysomnographic characteristics are presented for 110 implanted participants who underwent secondary outcome analyses; SD = standard deviation; Min = minimum; Max = maximum; BP = blood pressure.

Unscheduled Study Visit Type	n (%)
Clinic Visit with Medical Device Interrogation	60 (52.2%)
Awake Endoscopy	45 (39.1%)
Type I Polysomnography	33 (28.7%)
Drug-Induced Sleep Endoscopy	4 (3.5%)
Home Sleep Apnea Test	1 (0.9%)

Table S3. The number of unscheduled study visits that occurred during the study at the site investigators' discretion. n: number of subjects with at least one event, the same subject could have more than one event.

	SAF/FAS/PPS	Reason why data was excluded from the Per Protocol analysis
1	Y /N /N	Censored from analysis because study visitation inadvertently occurred at an unapproved site - only safety data analyzed
2	Y /N /N	Censored from analysis because study visitation inadvertently occurred at an unapproved site - only safety data analyzed
3	Y /N /N	Censored from analysis because study visitation inadvertently occurred at an unapproved site - only safety data analyzed
4	Y /N /N	Implant not successful
5	Y /N /N	Implant not successful
6	Y /Y /N	Withdrew consent after month 10
7	Y /Y /N	Withdrew consent after month 5
8	Y /Y /N	Withdrew after implanted device migration occurred
9	Y /Y /N	Major protocol deviation
10	Y /Y /N	12-month polysomnogram not completed due to device deficiency
11	Y /Y /N	Withdrew consent after month 5
12	Y /Y /N	Withdrew after implanted device extruded
13	Y /Y /N	Withdrew after unsuccessful implanted device revision surgery
14	Y /Y /N	Withdrew consent after month 10
15	Y /Y /N	Withdrew consent after month 5
16	Y /Y /N	Withdrew consent after month 9
17	Y /Y /N	Withdrew postoperatively due to dysphagia, device never activated

	SAF/FAS/PPS	Reason why data was excluded from the Per Protocol analysis
18	Y /Y /N	12-month visit not performed but continued in the study
19	Y /Y /N	Lost to follow up after month 9
20	Y /Y /N	Lost to follow up after month 3
21	Y /Y /N	Withdrew due to device migration noted at activation
22	Y /Y /N	12-month visit not performed but continued in the study
23	Y /Y /N	Lost to follow up after month 5
24	Y /Y /N	Month 12 polysomnogram not performed but month 12 visit performed
25	Y /Y /N	Non-compliance with the therapy
26	Y /Y /N	Lost to follow up after month 5
27	Y /Y /N	Withdrew after unsuccessful implanted device revision surgery

Table S4. Summary of reasons for exclusion of participants from the complete set of 115 participants who underwent safety and co-primary endpoint analyses (SAF; N=115) after bilateral hypoglossal nerve stimulator implantation. Participants with postoperative visit data were evaluated in a full analysis of primary and secondary outcomes (FAS; N=110). Ultimately 88 participants completed the study on a per-protocol basis (PPS).

Serious Adverse Event	Device Related	Procedure Related	Non-Device or Procedure Related
Atrial Fibrillation	-	-	1
Asthenia/hypoesthesia	-	-	2
Device extrusion	1	-	-
Device dislocation	2		-
Implant hematoma	-	1	-
Dysphagia/HGN paralysis	-	2	-
Left bundle branch block	-	1	-
Epistaxis		1	-

Table S5. Device- or implant procedure-related serious adverse events between surgery and the 12-month post-operative visit.

Non-Serious Adverse Events	m (n,%)
Unrelated to Device and Unrelated to Implant	**102 (54, 47.0%)**
Device-Related and/or Implant Procedure-Related	**210 (72, 62.6%)**
Device-Related	92 (44, 38.3%)
Implant Procedure-Related	106 (46, 40%)
Device and Implant Procedure-Related	12 (10, 8.7%)

Table S6. Non-serious adverse events adjudicated by the clinical events committee that occurred during the study, including bilateral hypoglossal nerve stimulation device- or implant procedure-related events. n: number of subjects with at least one event; m: number of events; the same subject could have more than one event.

Adverse Device Effects	Number of participants with events and causality				Percentage of resolved events
	Device-related	Procedure-related	Procedure and Device related	Total # Participants with Events (%)	
Application site irritation	21			21 (18.3%)	86%
Dysphagia	4	12	2	18 (15.7%)	89%
Incision site swelling	3	14		17 (14.8%)	100%
Medical device discomfort	10			10 (8.7%)	100%
Implant site hypoaesthesia		7		7 (6.1%)	86%
Procedural pain		7		7 (6.1%)	86%
Dysphonia	2	4		6 (5.2%)	100%
Glossodynia	5	1		6 (5.2%)	100%
Oropharyngeal pain	1	4	1	6 (5.2%)	100%
Post procedural contusion		6		6 (5.2%)	100%
Tongue movement disturbance		5		5 (4.3%)	100%
Tongue exfoliation	4			4 (3.5%)	100%
Cough	1	1	1	3 (2.6%)	100%
Glossitis	3			3 (2.6%)	100%
Odynophagia		3		3 (2.6%)	100%
Pain in jaw	2		1	3 (2.6%)	33%
Post procedural swelling		3		3 (2.6%)	67%
Speech disorder		3		3 (2.6%)	100%
Application site reaction	2			2 (1.7%)	100%
Dysarthria		2		2 (1.7%)	50%
Ear discomfort		2		2 (1.7%)	100%
Epistaxis		2		2 (1.7%)	100%
Headache	2			2 (1.7%)	100%
Hypoaesthesia oral	1	1		2 (1.7%)	100%
Implant site irritation		2		2 (1.7%)	100%
Incision site pain		2		2 (1.7%)	100%
Medical device pain	2		1	2 (1.7%)	100%
Neck pain	1		1	2 (1.7%)	100%

Adverse Device Effects	Number of participants with events and causality				Percentage of resolved events
	Device-related	Procedure-related	Procedure and Device related	Total # Participants with Events (%)	
Post procedural fever		2		2 (1.7%)	100%
Procedural headache		2		2 (1.7%)	100%
Procedural site reaction	1	1		2 (1.7%)	100%
Sleep disorder	1		1	2 (1.7%)	100%
Somnolence	1		1	2 (1.7%)	50%
Tinnitus	1	1		2 (1.7%)	50%
Tongue spasm	1	1		2 (1.7%)	100%
Ageusia		1		1 (0.9%)	100%
Anxiety		1		1 (0.9%)	100%
Application site infection		1		1 (0.9%)	100%
Application site ulcer	1			1 (0.9%)	100%
Back pain			1	1 (0.9%)	100%
Dermatitis contact		1		1 (0.9%)	100%
Dizziness	1			1 (0.9%)	100%
Dyspepsia	1			1 (0.9%)	100%
Ear pain	1			1 (0.9%)	50%
Fatigue		1		1 (0.9%)	100%
Foreign body		1		1 (0.9%)	100%
Gastroesophageal reflux disease	1			1 (0.9%)	0%
Hemorrhage	1			1 (0.9%)	100%
Hypoaesthesia		1		1 (0.9%)	100%
Implant site infection		1		1 (0.9%)	100%
Incision site hematoma		1		1 (0.9%)	100%
Ingrown hair		1		1 (0.9%)	100%
Jaw clicking		1		1 (0.9%)	100%
Jaw disorder	1			1 (0.9%)	100%
Muscle contractions involuntary	1			1 (0.9%)	100%
Musculoskeletal discomfort			1	1 (0.9%)	100%
Panic attack	1			1 (0.9%)	100%

Adverse Device Effects	Number of participants with events and causality				Percentage of resolved events
	Device-related	Procedure-related	Procedure and Device related	Total # Participants with Events (%)	
Paresthesia		1		1 (0.9%)	100%
Phlebitis		1		1 (0.9%)	100%
Post procedural diarrhea		1		1 (0.9%)	100%
Post procedural discomfort		1		1 (0.9%)	100%
Presyncope		1		1 (0.9%)	100%
Procedural nausea		1		1 (0.9%)	100%
Swelling	1			1 (0.9%)	100%
Swollen tongue	1			1 (0.9%)	100%
Tongue discomfort	1			1 (0.9%)	100%
Tooth disorder	1			1 (0.9%)	100%

Table S7. All non-serious adverse events that occurred during the study.

		Completers (N=89)	Non-Completers (N=26)	p-value	Total (N=115)
Sex	n	89	26		115
	Male	62 (69.7%)	19 (73.1%)	0.7372	81 (70.4%)
	Female	27 (30.3%)	7 (26.9%)		34 (29.6%)
Age (years)	n	89	26		115
	Mean ± SD	57.2 ± 6.8	55.5 ± 8.9	0.3150	56.8 ± 7.3
	Median	57	57		57
Race (more than one can be selected)	n	89	26		115
	American Indian or Alaska Native	0 (0.0%)	0 (0.0%)	1.000	0 (0.0%)
	Native Hawaiian or other Pacific Islander	0 (0.0%)	0 (0.0%)		0 (0.0%)
	Asian	0 (0.0%)	1 (3.8%)		1 (0.9%)
	White	83 (93.3%)	25 (96.2%)		108 (93.9%)
	Black or African American	4 (4.5%)	0 (0.0%)		4 (3.5%)
	Other	2 (2.2%)	0 (0.0%)		2 (1.7%)
Ethnicity	n	89	26		115
	Hispanic or Latino	7 (7.9%)	1 (3.8%)	0.0659	8 (7.0%)
	Not Hispanic or Latino	81 (91.0%)	22 (84.6%)		103 (89.6%)
	Not reported	1 (1.1%)	2 (7.7%)		3 (2.6%)
	Unknown	0 (0.0%)	1 (3.8%)		1 (0.9%)
Body Mass Index (kg/m²)	n	89	26		115
	Mean ± SD	28.64 ± 2.56	28.03 ± 2.85	0.3060	28.50 ± 2.63
	Median	28.8	28.5		28.7
AHI (events/hr)	n	89	23		112
	Mean ± SD	27.8 ± 11.5	29.5 ± 11.2	0.531	28.2 ± 11.4
	Median	24.3	28.8		25.7
Apnea Index (events/hr)	n	89	23		112
	Mean ± SD	11.6 ± 9.6	12.5 ± 10.3	0.6842	11.7 ± 9.7
	Median	9.2	10.0		15.4
Hypopnea Index (events/hr)	n	89	23		112
	Mean ± SD	16.3 ± 9.3	17.0 ± 8.3	0.7234	28.6 ± 12.9
	Median	14.8	16.7		26.6
Supine AHI (events/hr)	n	89	23		112
	Mean ± SD	49.4 ± 20.7	48.5 ± 14.8	0.8563	49.2 ± 19.6
	Median	49.7	49.3		49.5
Non Supine AHI (events/hr)	n	89	23		112
	Mean ± SD	11.8 ± 11.4	16.1 ± 16.5	0.2490	12.6 ± 12.7
	Median	7.8	12.0		9.1
REM AHI (events/hr)	n	89	23		112
	Mean ± SD	23.7 ± 17.6	29.1 ± 16.9	0.1871	24.8 ± 17.5
	Median	18.8	24.4		21.1
NREM AHI (events/hr)	n	89	23		112
	Mean ± SD	28.5 ± 13.2	28.9 ± 12.00	0.9135	28.6 ± 12.9

		Completers (N=89)	Non-Completers (N=26)	p-value	Total (N=115)
	Median	25.3	28.8		26.6

Table S8. Comparison of baseline demographic, anthropomorphic, and polysomnographic variables between participants who completed the 12-month polysomnogram and those who did not. n: number of subjects within the specified group. AHI: apnea-hypopnea index. SD: standard deviation.

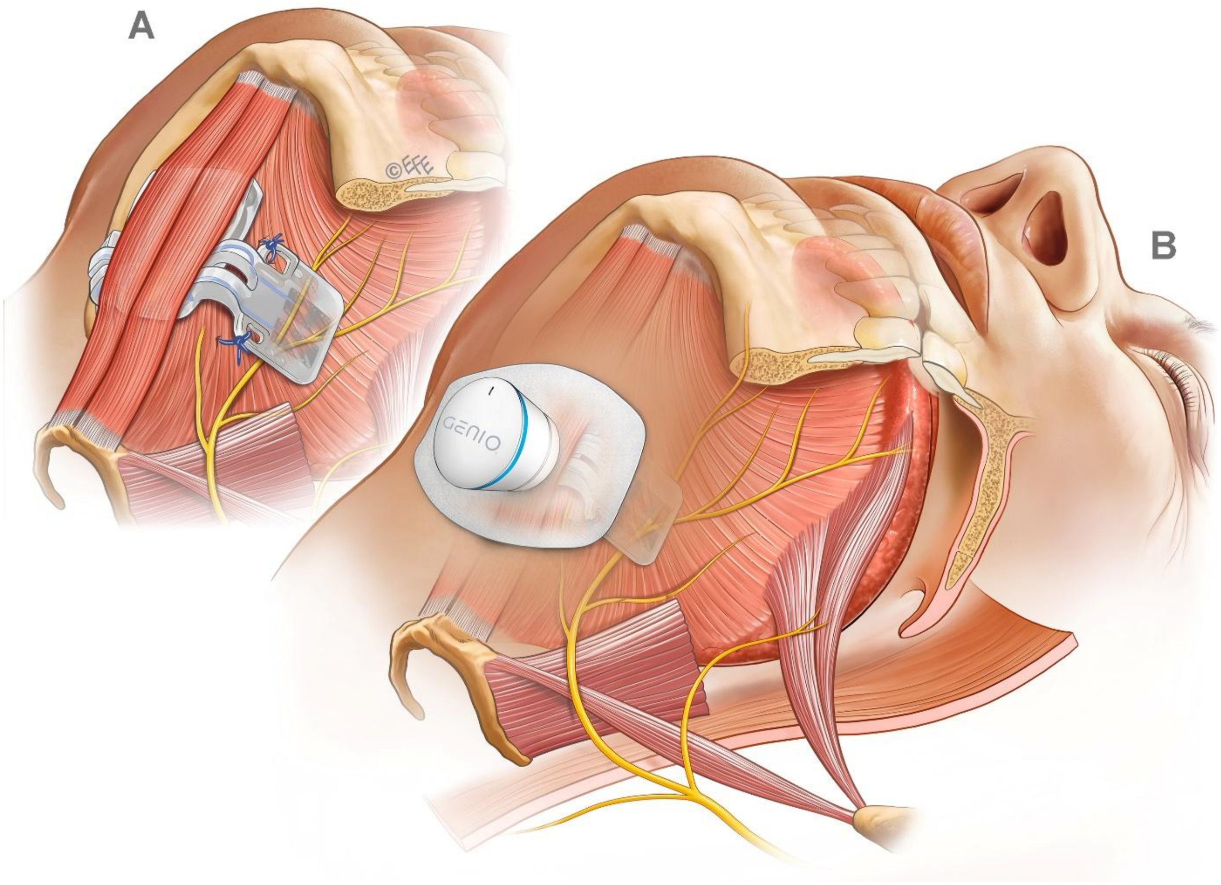

Figure S1. A) The single-component bilateral hypoglossal nerve stimulation device implant straddles the genioglossus muscles and activates the protrusor branches of the hypoglossal nerve. B) The activation unit attaches to a disposable patch that is applied externally before sleep.

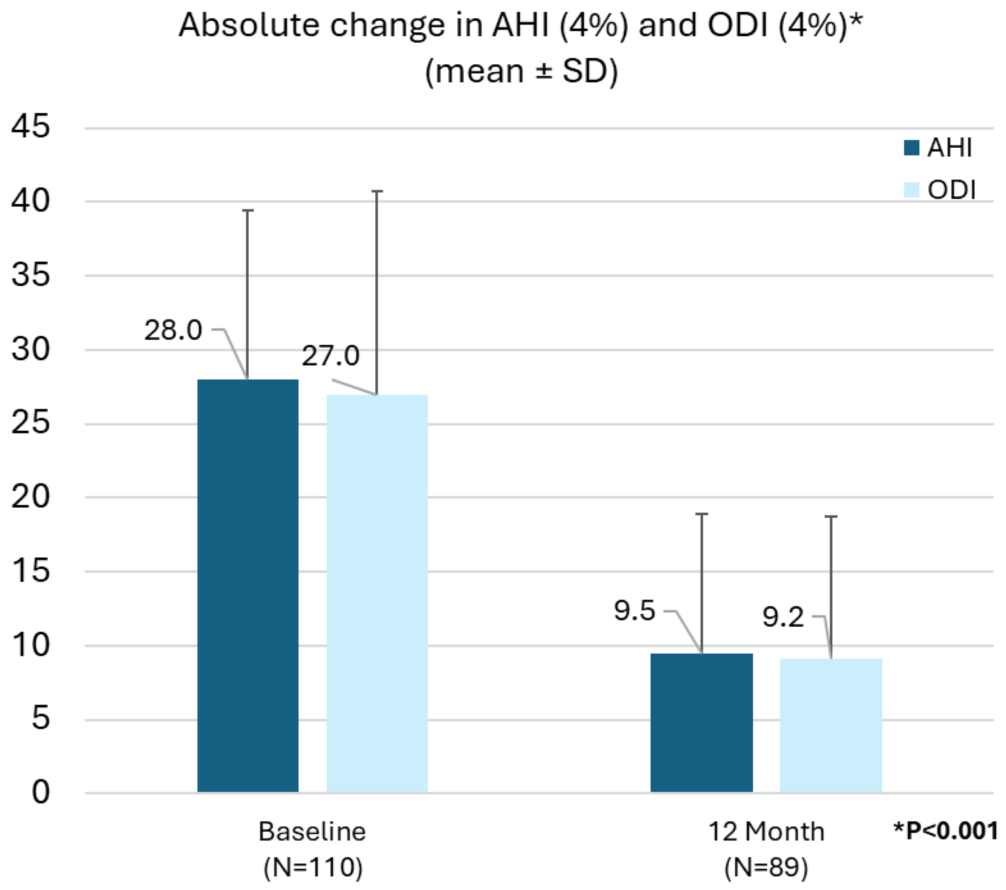

Figure S2. The absolute change in apnea-hypopnea index (AHI) and oxygen desaturation index (ODI) in all participants with available data from baseline to 12 months postoperatively after bilateral hypoglossal nerve stimulator implantation.

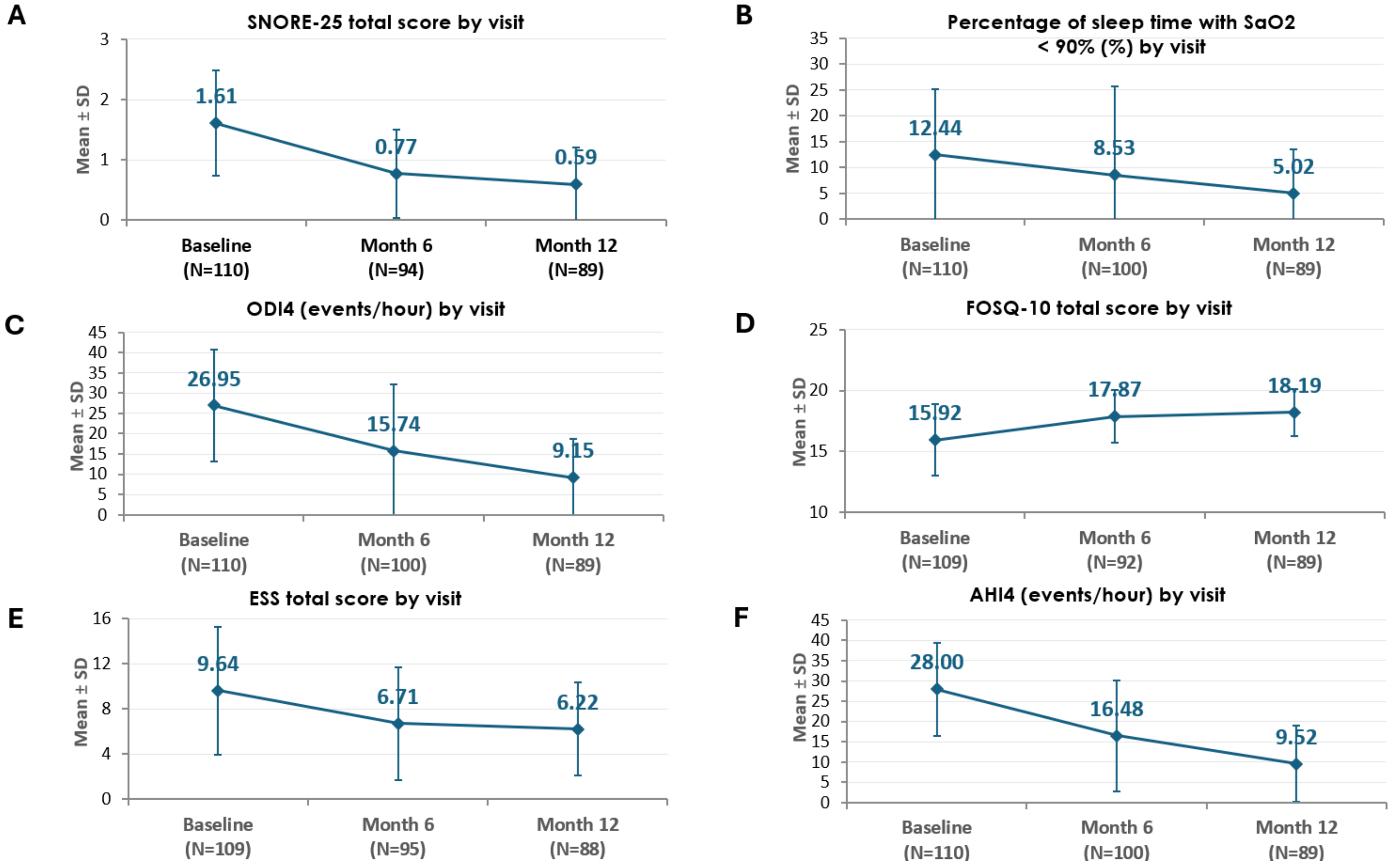

Figure S3. Secondary outcomes from baseline to 6- and 12-months after bilateral hypoglossal nerve stimulator implantation. AHI4 = 4% apnea-hypopnea index; ODI4 = 4% oxygen desaturation index; ESS = Epworth Sleepiness Scale; SaO2 = blood oxygen saturation; FOSQ-10 = Functional Outcomes of Sleep Questionnaire Short Form.

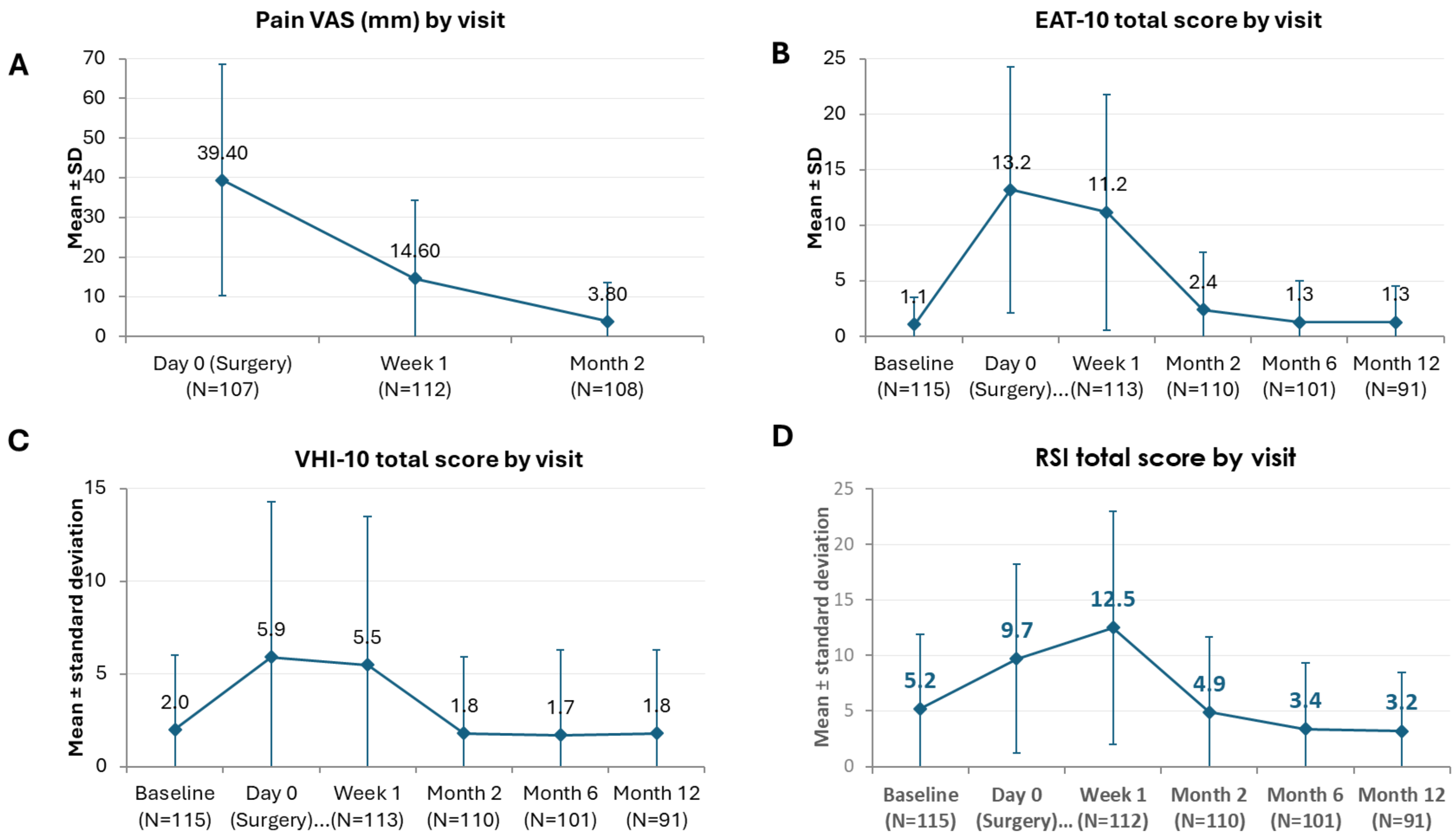

Figure S4. Secondary outcomes from baseline up to 12-months after bilateral hypoglossal nerve stimulator implantation. VAS = Visual Analog Scale; EAT-10 = Eating Assessment Tool; VHI-10 = Voice Handicap Index-10; RSI = Reflux Symptom Index.

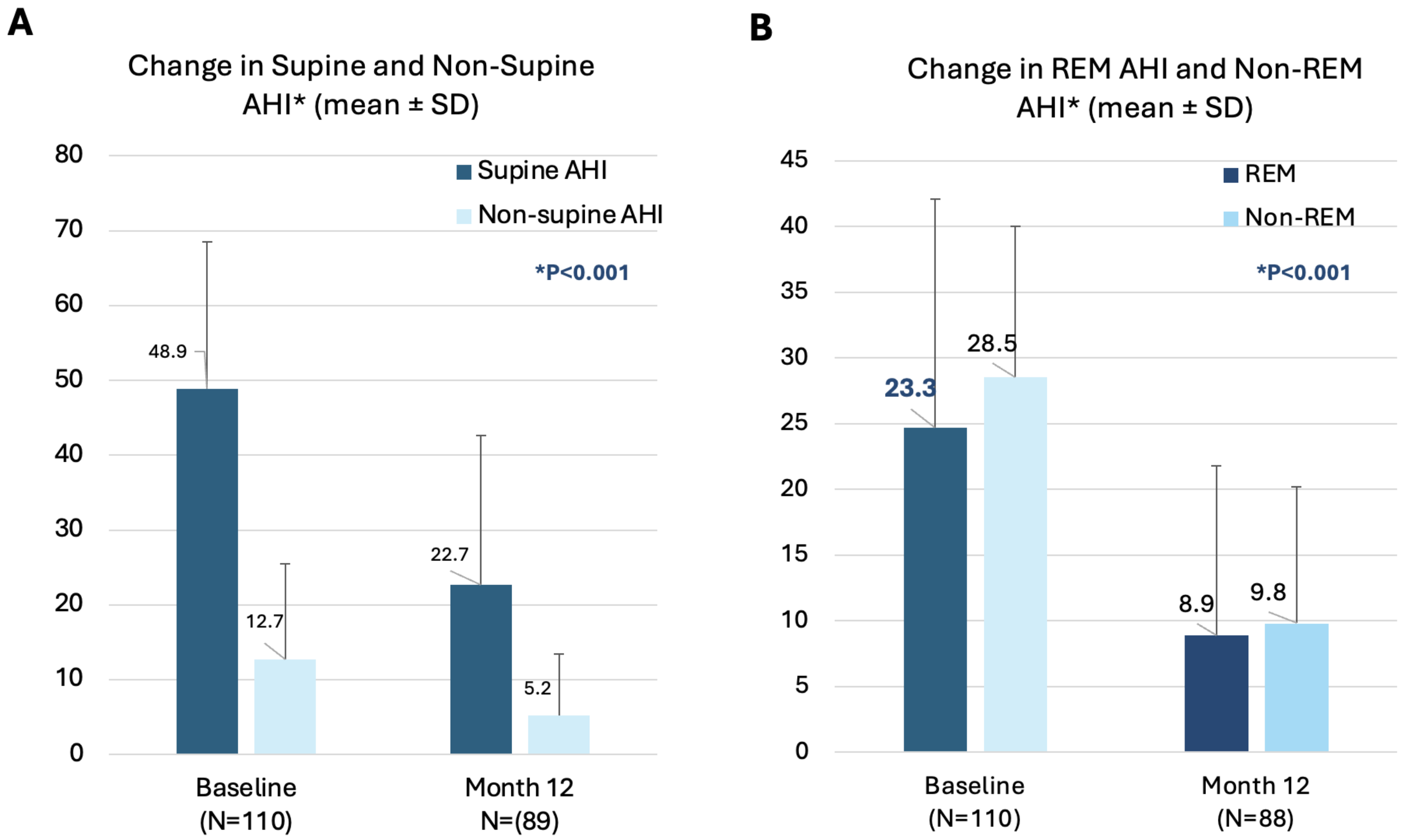

Figure S5. **A**. Changes in supine- and non-supine apnea-hypopnea index (AHI) from baseline to 12 months after bilateral hypoglossal nerve stimulator implantation. **B**. Changes in REM and non-REM AHI from baseline to 12 months after bilateral hypoglossal nerve stimulator implantation.

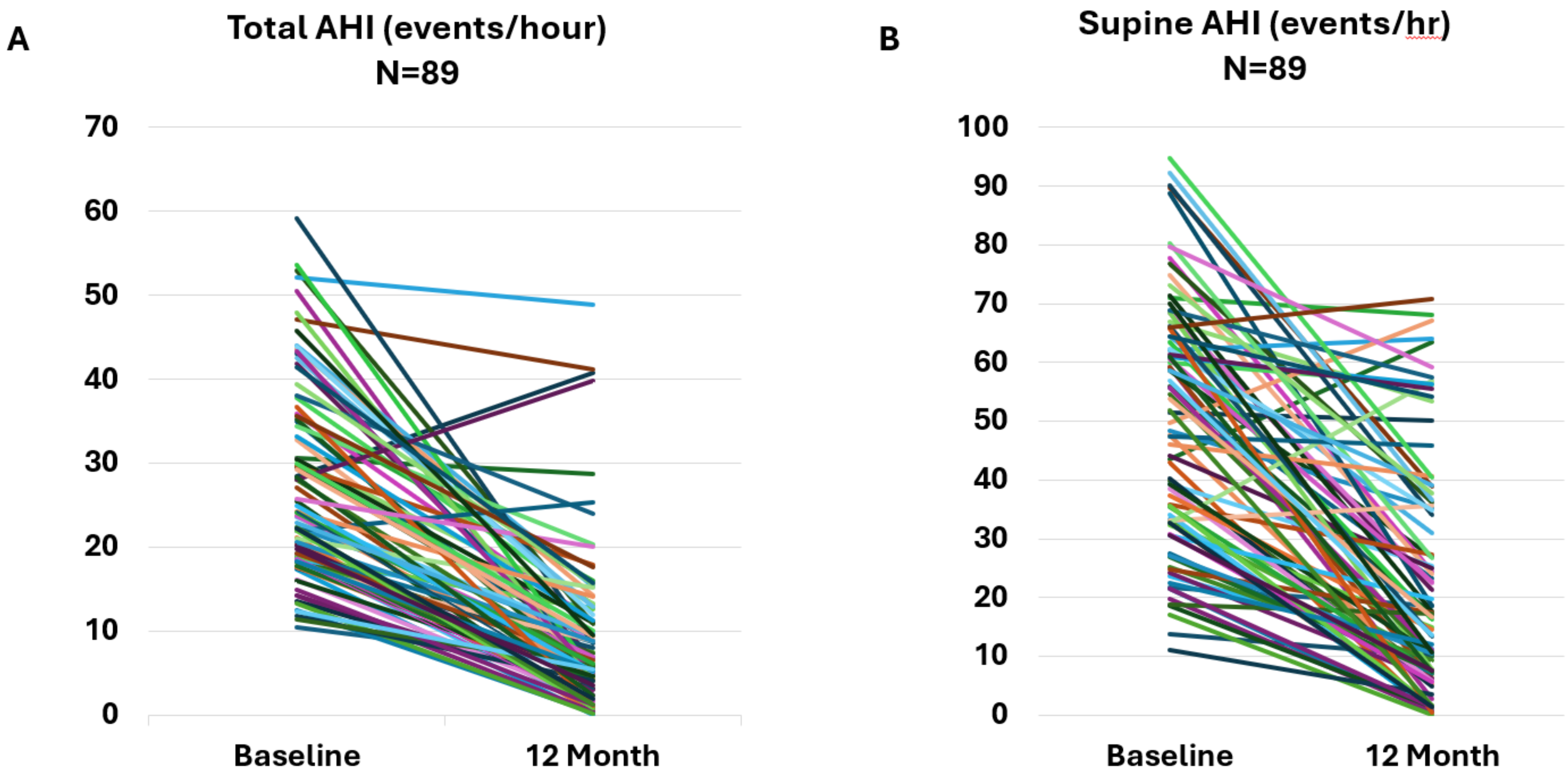

Figure S6. **A**. Per-participant changes in total apnea-hypopnea index (AHI) from baseline to 12 months after bilateral hypoglossal nerve stimulator implantation. **B**. Per-participant changes in supine AHI from baseline to 12 months after bilateral hypoglossal nerve stimulator implantation.

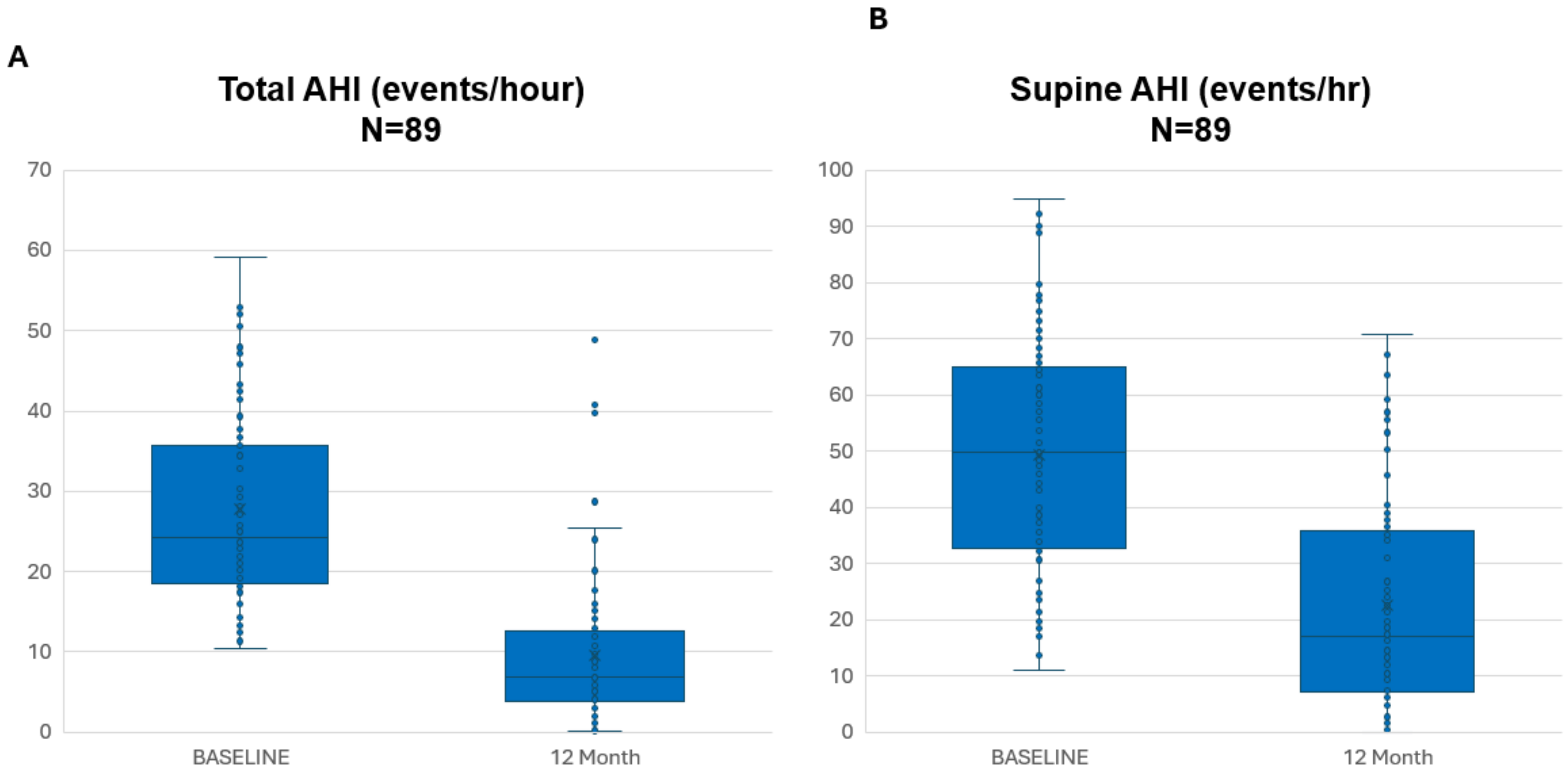

Figure S7. **A**. Per-protocol participant changes in total apnea-hypopnea index (AHI) from baseline to 12 months after bilateral hypoglossal nerve stimulator implantation. **B**. Per-protocol participant changes in supine AHI from baseline to 12 months after bilateral hypoglossal nerve stimulator implantation.
